# *Ureaplasma* Species in Perinatal Disease: From the Age of Innocence to the Missing Villain

**DOI:** 10.3390/ijms27156865

**Published:** 2026-07-31

**Authors:** Vlad Dima, Andreea Calomfirescu Avramescu, Andrada Mirea, Adrian Ioan Toma, Roxana-Elena Bohiltea, Anca Bivoleanu, Dan Lee Stewart

**Affiliations:** 1 Department of Neonatology, Spitalul Clinic Filantropia, 011171 Bucharest, Romania; vlad.dima@spitalulfilantropia.ro (V.D.);; 2 Balneophysiokinesitherapy and Rehabilitation Department, Carol Davila University of Medicine and Pharmacy, 050474 Bucharest, Romania; 3 Faculty of Medicine, Titu Maiorescu University, 040051 Bucharest, Romania; 4 Cuza Voda Hospital, 700038 Iasi, Romania; 5 Norton Children’s Hospital, University of Louisville School of Medicine, Louisville, KY 40202, USA

**Keywords:** *Ureaplasma urealyticum*, *Ureaplasma parvum*, preterm birth, chorioamnionitis, bronchopulmonary dysplasia, intraventricular hemorrhage, neonatal sepsis, vertical transmission, azithromycin, neonatal neuroinflammation, NF-κB signaling, NLRP3 inflammasome, blood–brain barrier, Toll-like receptor, multiple-banded antigen, biofilm, macrolide therapy, serovar virulence, metagenomic sequencing, conditional pathogenicity

## Abstract

*Ureaplasma urealyticum* and *Ureaplasma parvum* occupy an odd place in perinatal medicine: dismissed for decades as harmless residents of the female genital tract, they are now recognized as pathogens with real consequences for preterm newborns. This review traces that paradigm shift, from organisms once dismissed as harmless colonizers to pathogens now implicated in chorioamnionitis, preterm birth, and a range of serious neonatal morbidities, and describes the molecular mechanisms that underlie their pathogenicity: Toll-like receptor (TLR1/2/6/9)-mediated NF-κB and MyD88/IRAK4/TRAF6 signaling, NLRP3 inflammasome activation and pyroptosis, and blood–brain barrier disruption via claudin-5/occludin downregulation and MMP-mediated tight junction cleavage. We also review the evidence for biofilm-conferred antibiotic tolerance and the clinical associations between *Ureaplasma* colonization and intraventricular hemorrhage (pooled OR 1.62, 95% CI 1.23–2.13), bronchopulmonary dysplasia (pooled OR 2.30, 95% CI 1.65–3.20), late-onset sepsis, and neurodevelopmental impairment. Diagnosis remains a weak point: culture sensitivity is below 10% compared with polymerase chain reaction (PCR) testing, and no randomized trial has yet shown that microbiological eradication translates into better clinical outcomes—a gap we examine critically. Whether these organisms cause disease seems to depend on gestational age, bacterial load, serovar-specific virulence, and host immune competence. We argue that this conditionality calls for risk stratification rather than dismissal whenever *Ureaplasma* is identified in clinical specimens, and that the field needs a paradigm shift toward *Ureaplasma* screening in high-risk pregnancies and targeted neonatal PCR testing, backed by adequately powered interventional trials.

## 1. Introduction

Few organisms illustrate the gap between clinical dismissal and scientific concern as well as the human *Ureaplasma* species. *Ureaplasma urealyticum* (serovars 2, 4, 5, and 7–13) and *Ureaplasma parvum* (serovars 1, 3, 6, and 14), classified within the class Mollicutes and family Mycoplasmataceae, are among the smallest self-replicating organisms known to exist independently [1] (a serovar being a distinct variant within a species). What defines them biochemically is their ability to hydrolyze urea to generate adenosine triphosphate—the metabolic trait that gave the genus its name, and one that also produces ammonia capable of causing localized cytotoxic injury [2,3]. Because they have no cell wall, they are naturally resistant to the antibiotic classes that target cell-wall synthesis, which makes the empirical regimens routinely used in neonatal intensive care units largely useless against them [4].

*Ureaplasma* organisms are detectable in the vaginal flora of 40–80% of healthy reproductive-age women, and colonization becomes even more common during pregnancy [5]. This sheer prevalence shaped the dominant clinical narrative for decades: *Ureaplasma* spp. were treated as benign commensals—passengers in the reproductive tract rather than agents of disease. Early studies reinforced this view. Because *T-mycoplasma* (an older name reflecting the tiny colonies it forms in culture) was so often isolated from fertile women, its frequent presence was read as proof of harmlessness rather than as a clue worth investigating further [6,7,8].

That narrative has since been substantially revised. As molecular diagnostics capable of detecting non-culturable organisms matured, and as animal models of intrauterine infection and large prospective cohorts accumulated, a very different picture emerged. *Ureaplasma* spp. are now recognized as the bacteria most commonly isolated from infected amniotic fluid in preterm pregnancies, with a documented causal link to chorioamnionitis, preterm premature rupture of membranes, and spontaneous preterm delivery [9,10]. In the preterm neonate—particularly the extremely low birth weight infant born before 28 weeks—*Ureaplasma* spp. are the pathogens most frequently identified in respiratory secretions, blood, and cerebrospinal fluid [11].

Yet the story remains incomplete. Not every woman colonized by *Ureaplasma* spp. delivers prematurely, and not every colonized preterm infant develops bronchopulmonary dysplasia, intraventricular hemorrhage, or neurodevelopmental sequelae. Whether these organisms cause disease appears to depend on several converging factors: the virulence of the specific serovar, the bacterial burden, how long the intrauterine exposure lasts, the gestational age at infection, the integrity of cervical and immune barriers, and the presence of other colonizing microorganisms [12,13]. This conditional nature has made it difficult to establish clear-cut causality, and it has slowed the translation of the microbiological evidence into screening and treatment guidelines.

The title of this review is deliberately provocative. “From the age of innocence to the missing villain” is not a rhetorical flourish but a statement of where the evidence now stands: *Ureaplasma* spp. sit squarely within the causal pathway of some of prematurity’s most devastating complications, and yet they remain largely absent from routine diagnostic and therapeutic protocols in neonatal medicine. Our aim here is to trace the historical trajectory of *Ureaplasma* research, lay out the mechanisms by which these organisms cause harm, characterize their clinical associations precisely, and make the case for revisiting current practice in obstetric and neonatal care.

## 2. Literature Search Strategy

**Figure 1:**
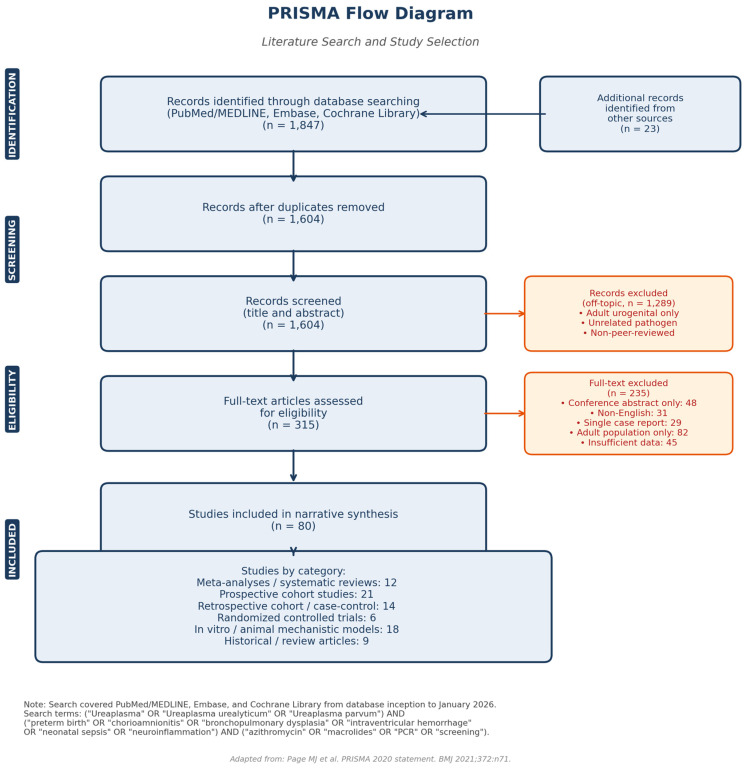
Flow diagram of the literature search and selection process, presented in a format adapted from the PRISMA 2020 statement for illustrative purposes. Records were identified through searches of PubMed/MEDLINE, Embase, and Cochrane Library (inception to January 2026). As this is a narrative rather than a systematic review, the full PRISMA methodology (protocol registration, dual independent screening, formal risk-of-bias assessment) was not followed; the diagram format is used here only to present the search and selection process transparently. Format adapted from: Page MJ et al. The PRISMA 2020 statement. BMJ 2021;372:n71 [14].

To synthesize the literature on the evolving role of *Ureaplasma* species in perinatal and neonatal medicine as comprehensively and objectively as possible, we conducted a structured search of PubMed/MEDLINE, Embase, and the Cochrane Library. The search covered articles from database inception through January 2026, with particular attention to molecular epidemiological data and clinical trials published between 2020 and 2025. It should be noted that this is a narrative review rather than a formal systematic review: we did not register a protocol, perform dual independent screening, or carry out a formal risk-of-bias assessment. The search and selection process is nonetheless summarized visually, in a format adapted from PRISMA, purely for the sake of transparency (Figure 1).

The search combined Medical Subject Headings (MeSH) terms with free-text keywords, adapted to each database using Boolean operators. The core architecture was: (“*Ureaplasma*” OR “*Ureaplasma urealyticum*” OR “*Ureaplasma parvum*” OR “*T-mycoplasma*”) AND (“preterm birth” OR “chorioamnionitis” OR “bronchopulmonary dysplasia” OR “intraventricular hemorrhage” OR “neonatal sepsis” OR “neuroinflammation” OR “vertical transmission”) AND (“azithromycin” OR “macrolides” OR “polymerase chain reaction” OR “screening”).

Inclusion criteria: we included controlled clinical trials, prospective and retrospective cohort studies, case–control studies, systematic reviews, meta-analyses, and well-characterized animal or in vitro mechanistic studies addressing *Ureaplasma* spp. pathogenicity, diagnostics, or treatment in obstetric or neonatal populations. Only English-language articles were included.

Exclusion criteria: we excluded studies limited to adult urogenital infection unrelated to pregnancy, single case reports (except where they documented genuinely novel presentations, such as atypical chronic meningitis), conference abstracts without accessible full texts, and non-peer-reviewed commentaries.

In total, 80 key references were retained, selected for scientific rigor, sample size, historical relevance, and direct bearing on the clinical and molecular outcomes discussed in this review.

Because this is a narrative rather than a systematic review, several limitations inherent to the format should be made explicit. Selection bias is possible: although we aimed for comprehensiveness, the choice of which studies to foreground reflects the authors’ clinical and mechanistic priorities rather than a pre-registered, reproducible algorithm, and dual independent screening was not performed. Citation bias is a related concern—studies reporting statistically significant or mechanistically striking findings tend to be cited and re-cited more often than negative or null studies, which can inflate the apparent strength and consistency of an association. We have tried to counteract this by explicitly retaining and discussing negative or inconclusive studies (for example, the single-center cohorts that failed to confirm an association with intraventricular hemorrhage, and the non-significant pooled estimate for necrotizing enterocolitis) rather than omitting them. We did not apply a formal quality-weighting scheme, such as GRADE, to individual studies, so associations drawn from small, single-center, or retrospective series are not textually distinguished from those drawn from larger meta-analyses; study design and sample size are indicated, where available, in Table 1, Table 2 and Table 3, and readers should weigh each citation accordingly. These limitations do not undermine the mechanistic and clinical case assembled here, but they mean this review should be read as an argument built on the best currently available evidence, not as a substitute for a registered systematic review or a clinical practice guideline.

The complete search and selection process is shown in Figure 1.

**Figure 2:**
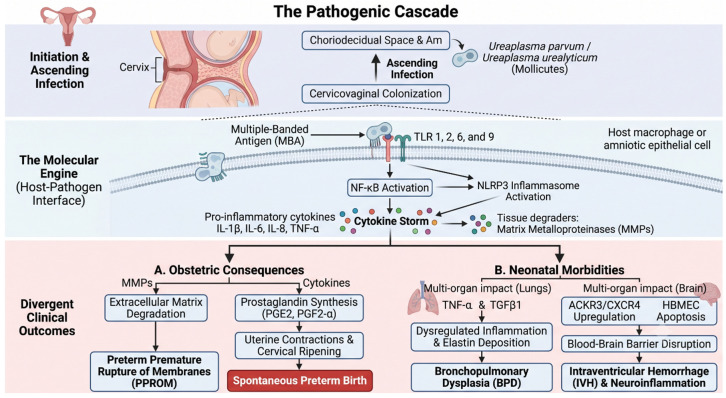
The pathogenic mechanisms of *Ureaplasma* spp. in preterm birth and neonatal morbidity. Ascending infection from the lower genital tract introduces *Ureaplasma* into the amniotic cavity. The bacterial Multiple-Banded Antigen (MBA) engages host Toll-like receptors (TLR1/2/6/9), triggering NF-κB activation and NLRP3 inflammasome activation. This results in a localized cytokine cascade (IL-1β, IL-6, IL-8, TNF-α) and the release of matrix metalloproteinases (MMPs). In the obstetric compartment, MMPs degrade fetal membranes, causing rupture, while prostaglandins drive uterine contractions and preterm delivery. In the susceptible preterm neonate, systemic inflammation disrupts the blood–brain barrier, contributing to intraventricular hemorrhage risk on top of the germinal-matrix fragility that remains its principal cause, and promotes fibrotic lung injury characteristic of bronchopulmonary dysplasia.

The path by which *Ureaplasma* spp. move from harmless commensal of the lower genital tract to driver of preterm birth and neonatal organ injury is now understood at the molecular level, and it unfolds in several steps (Figure 2). Infection ascends from the cervicovaginal space into the choriodecidual and amniotic compartments, where the organism’s surface Multiple-Banded Antigen (MBA) lipoprotein engages host Toll-like receptors, chiefly TLR2/6 and TLR9, triggering MyD88-dependent NF-κB activation and assembly of the NLRP3 inflammasome. The resulting cytokine cascade (IL-1β, IL-6, IL-8, TNF-α) and release of matrix metalloproteinases (MMPs) act on two fronts at once. In the obstetric compartment, MMPs break down the extracellular matrix of the fetal membranes while prostaglandins E2 and F2-α drive uterine contractions, together producing preterm premature rupture of membranes and spontaneous preterm delivery. In the preterm neonate born into this inflammatory environment, the same cytokines promote fibrotic remodeling of the lung through TGF-β1 signaling, the hallmark of bronchopulmonary dysplasia, while systemic inflammation compromises blood–brain barrier integrity by downregulating tight junction proteins and through MMP-mediated cleavage, laying the groundwork for a contribution to intraventricular hemorrhage risk and white matter injury, on top of the germinal-matrix fragility that is the principal driver of IVH in extreme prematurity. This sequence is not inevitable: gestational age, bacterial burden, serovar virulence, and host immune competence all shape whether it proceeds, which is why colonization is nearly universal but overt disease is not—a distinction that forms the central argument of this review.

## 3. Historical Perspective: *T-Mycoplasma* and the Early Literature

The discovery of *Ureaplasma urealyticum*—then called *T*-*mycoplasma* for the characteristically tiny colonies it forms on agar—dates to Shepard’s 1954 report of its isolation from patients with nongonococcal urethritis [24]. For roughly the next two decades, clinical interest in the organism stayed modest, partly because culture was slow and technically demanding, and partly because it colonized so many healthy people that attributing disease to it was far from straightforward.

A turning point came in 1972, when Gnarpe and Friberg reported that *T-mycoplasma* was isolated far more often from the cervical secretions of infertile women than from fertile pregnant controls, and proposed a role in reproductive failure [25]. The finding launched a decade of research that proved productive but ultimately inconclusive. Friberg and Gnarpe went on to track pregnancy rates after doxycycline treatment, and Kundsin reported conception rates of 46% at three months and 84% at one year following tetracycline therapy for unexplained infertility [26]. Idriss and colleagues, studying 200 consecutive infertility patients, found significantly more positive cultures among women with unexplained infertility (55%) than among controls (32%), yet still concluded that the organism’s causal role was “neither proven nor disproven” [6]. Rehewy and colleagues reported a similarly modest excess of isolation in infertile women and found no effect on sperm penetration in vitro—evidence that, at the time, reinforced the commensalist view [7].

Meanwhile, obstetric researchers were accumulating their own observations linking *T-mycoplasma* to reproductive failure more broadly. Kundsin and Driscoll had already proposed links to low birth weight, spontaneous abortion, and infertility as early as 1970. By the early 1980s, Eschenbach’s model of polymicrobial pelvic inflammatory disease explicitly placed genital *mycoplasmas*—*Ureaplasma* included—among the putative initiators of lymphatic and perivascular inflammation around the uterus, even without direct infection of the fallopian tubes [27]. Eschenbach also identified *Mycoplasma* as the sole endometrial and blood isolate in roughly 15% of women with postpartum endometritis, adding a further dimension to the clinical picture [27].

The link to neonatal morbidity emerged more gradually. *T-mycoplasma* was first isolated from cord blood in 1969, and by the mid-1970s, investigators were proposing a role in neonatal respiratory disease after recovering the organism from the lungs of stillborn infants with pneumonitis. By 1988, three separate research groups had published cohort studies connecting *Ureaplasma* airway colonization to chronic lung disease in very-low-birth-weight infants, and a meta-analysis of seventeen studies published before 1995 confirmed a significant association with bronchopulmonary dysplasia [28]. The organism had moved from reproductive curiosity to putative neonatal pathogen—yet the “missing villain” remained just out of reach, its role obscured again and again by inconsistent methods, uneven diagnostics, and the sheer biological complexity of extreme prematurity.

Matters grew more complicated still when, in 2002, the original fourteen serovars were formally reclassified into two distinct species, *U. urealyticum* and *U. parvum* [29]. Many earlier studies had never distinguished between the two, and it later became clear that *U. parvum* was by far the more prevalent species in clinical samples, particularly in the amniotic cavity, while questions about serovar-specific virulence remained—and remain—an active area of investigation [30].

## 4. Microbiology, Taxonomy, and Virulence Factors

*Ureaplasma* spp. belong to the order Mycoplasmoidales, family Mycoplasmataceae, and class Mollicutes, and rank among the smallest free-living, self-replicating prokaryotes known, with genomes of roughly 580,000 to 840,000 base pairs [1,31]. Lacking a cell wall entirely—the defining feature of the Mollicutes—leaves them intrinsically resistant to every β-lactam and glycopeptide antibiotic, and it is also why they are so hard to culture: growth requires specialized media supplemented with urea, serum, and additional growth factors [4,32].

The fourteen recognized serovars now fall under two species: *U. parvum* (biovar 1; serovars 1, 3, 6, and 14) and *U. urealyticum* (biovar 2; serovars 2, 4, 5, and 7–13) [29]. Across all of them, certain features recur—limited biosynthetic capacity, dependence on host-derived lipids and cholesterol, a preference for mucosal tissue, and expression of the multiple-banded antigen (MBA). This surface lipoprotein is the dominant pathogen-associated molecular pattern the host immune system recognizes, and it is generally regarded as the leading candidate virulence factor [33,34].

MBA is a diacylated lipoprotein recognized by Toll-like receptors 1, 2, 6, and 9, and its engagement activates NF-κB signaling and the downstream upregulation of proinflammatory cytokines [34,35]. Mutations in the mba gene drive phase and size variation of MBA, giving the organism a way to evade host immune surveillance—a mechanism demonstrated both in vitro and in animal models of chronic intrauterine infection [33,36]. A second major virulence mechanism centers on urease, the enzyme that hydrolyzes urea into ammonia and carbon dioxide. The ammonia produced is directly cytotoxic to epithelial surfaces, disrupts mucosal barrier integrity, and likely contributes to tissue injury in the preterm neonate’s respiratory tract and central nervous system [2,3,37].

IgA proteases and phospholipases A and C have historically been credited to *Ureaplasma* spp. in functional assays, but genomic analysis of all fourteen serovars found no coding sequences homologous to known IgA protease or phospholipase C genes—casting real doubt on these proposed mechanisms [38]. Phospholipase A activity, however, has been demonstrated functionally and remains a plausible contributor to membrane degradation and the onset of preterm labor, given its relevance to prostaglandin synthesis [37]. *Ureaplasma* spp. can also form biofilms in vitro, a property that may shield the organism from both host defenses and antibiotics and that likely underlies the chronic, persistent nature of intrauterine colonization [39].

### 4.1. Biofilm Formation: Molecular Architecture and Clinical Consequences

Biofilm formation is an underappreciated but clinically important virulence strategy in *Ureaplasma* spp. Pandelidis and colleagues showed that both *U. urealyticum* and *U. parvum* form structured biofilms on polystyrene and on human epithelial cells in vitro, and that biofilm-forming isolates tolerate markedly higher concentrations of azithromycin and ciprofloxacin than their free-living (planktonic) counterparts [39]. This tolerance likely arises through several overlapping mechanisms: the extracellular polymeric substance (EPS) matrix restricts drug diffusion, sessile bacteria shift into an altered metabolic state, and a subpopulation of persister cells survives concentrations that would otherwise be lethal. The molecular composition of the *Ureaplasma* EPS matrix is not yet fully characterized, though genomic evidence points to surface lipoproteins—including MBA variants—as contributors to initial adhesion and matrix scaffolding. No formal quorum-sensing system has been found for *Ureaplasma*, owing to its markedly reduced genome; however, density-dependent MBA phase variation, which has been documented in models of chronic intra-amniotic infection, may serve a similar regulatory function [36]. The clinical stakes are considerable. Standard susceptibility testing, performed on planktonic organisms, may substantially underestimate the drug concentrations needed to eradicate *Ureaplasma* in vivo—one likely reason macrolide regimens sometimes fail to clear respiratory colonization in preterm infants. Biofilms forming directly on endotracheal tube surfaces in ventilated neonates create a protected niche for ongoing lung colonization, shielded from systemic drug concentrations. And within the amniotic cavity, persistent biofilm colonization can sustain low-grade inflammation for weeks without producing overt clinical signs—precisely the pattern described in prospective chorioamnionitis studies [40,41].

### 4.2. TLR Signaling Cascade, NLRP3 Inflammasome Activation, and Blood-Brain Barrier Disruption

MBA engages TLR2/6 as a heterodimer recognizing diacylated lipopeptides, and TLR9 via unmethylated CpG DNA motifs released during bacterial replication, on innate immune cells in the fetal membranes and choriodecidua [34,35]. TLR2/6 engagement recruits MyD88, which assembles the Myddosome and activates IRAK4 and IRAK1; this leads to TRAF6 ubiquitination and, through the IKK complex, phosphorylation and proteasomal degradation of IκBα. NF-κB—predominantly the p65/p50 heterodimer—is thereby released to translocate to the nucleus, where it drives transcription of IL-1β, IL-6, IL-8, TNF-α, and cyclooxygenase-2 [34]. In parallel, TIRAP-dependent signaling activates the MAPK cascades (ERK1/2, p38, JNK), further amplifying AP-1-driven cytokine transcription. Together, these pathways generate a fast, self-reinforcing pro-inflammatory state within the amnion and chorion.

NLRP3 inflammasome activation is a molecularly distinct and particularly damaging arm of this response. In primate models of choriodecidual inoculation, *U. parvum* infection triggers NLRP3 assembly through the classic activating signals: potassium efflux from membrane disruption caused by urease-generated ammonia, mitochondrial reactive oxygen species arising from metabolic stress, and lysosomal damage from phagocytosed bacterial components [42]. Assembled NLRP3 recruits ASC and procaspase-1 to form the active inflammasome, which cleaves procaspase-1 into caspase-1. Active caspase-1 processes pro-IL-1β and pro-IL-18 into their mature forms and cleaves gasdermin D, whose N-terminal fragment then oligomerizes into pores in the plasma membrane—the molecular event underlying pyroptosis. This lytic form of cell death spills the cell’s full contents, including damage-associated molecular patterns, into the surrounding tissue, setting off a cycle of sterile inflammation that can persist even after the bacteria themselves are cleared. Notably, NLRP3 activation has been observed in fetal membranes before intra-amniotic infection is even established, suggesting that choriodecidual infection alone is enough to prime the inflammasome pathway [42].

Blood–brain barrier disruption by *Ureaplasma* spp. follows a molecular route distinct from that of classical bacterial meningitis pathogens. In human brain microvascular endothelial cell models, *Ureaplasma* spp. upregulate ACKR3 and CXCR4 while increasing VEGF expression, which together destabilize tight junction complexes and raise paracellular permeability [43]. The tight junction proteins claudin-5 and occludin—which, along with ZO-1, form the BBB’s molecular seal—are downregulated at the transcriptional level, while MMP-2 and MMP-9 are released into the surrounding space to cleave these junction proteins and degrade the basement membrane beneath them [43]. At the same time, *Ureaplasma* spp. induce endothelial apoptosis through the intrinsic caspase cascade (caspases 3, 7, and 9), while paradoxically suppressing caspases 1 and 4, which would otherwise help coordinate an effective innate immune response in the CNS [44]. This combination—a barrier that lets bacteria and cytokines through, paired with locally suppressed inflammatory clearance—helps explain the clinically paradoxical picture of chronic *Ureaplasma* meningitis without the classical CSF pleocytosis, as seen in the eight-month meningitis case described below [45].

At the population level, *U. parvum* predominates in clinical samples, accounting for the majority of amniotic fluid, placental, and neonatal respiratory isolates across most series [30,46]. Among its serovars, serovar 3 and serovar 6 have shown the most consistent association with adverse pregnancy outcomes in prospective studies, and vaginal colonization with serovar 3 in early pregnancy has emerged as an independent risk factor for spontaneous preterm birth in a prospective multicenter cohort [47].

## 5. Epidemiology of Colonization, Vertical Transmission, and Risk Stratification

Colonization of the female lower genital tract with *Ureaplasma* spp. varies by population and detection method but generally falls between 40% and 80% of women of reproductive age [5]. Sexual activity, hormonal status, coexisting bacterial vaginosis, and immune status all influence this figure, and prevalence rises further as pregnancy progresses. Because PCR detects non-viable organisms and offers far greater analytical sensitivity than culture, PCR-based estimates run substantially higher—in some series, culture missed more than 90% of PCR-positive specimens [48].

Among pregnant women, colonization rates as high as 82% have been reported [49]. Vertical transmission to the neonate can occur through ascending intrauterine infection, passage through a colonized birth canal, or hematogenous spread across the placenta [50]. Gestational age at delivery strongly influences transmission: very-low-birth-weight infants (under 1500 g) show respiratory tract colonization rates of 20–45%, and infants under 1000 g approach 90% colonization in some series [11,51]. One prospective study using cord blood cultures found *Ureaplasma* spp. in 23% of samples from infants born between 23 and 32 weeks, and a later report found the organism in 23.6% of serum or CSF PCR samples from very-low-birth-weight infants [11,52].

Colonization rates peak within the first week after birth, consistent with acquisition during delivery. In a large Chinese retrospective study of 7257 neonates, the overall PCR detection rate was 7.73%, highest among extremely preterm and vaginally delivered infants [18]. Vaginal delivery carries a higher transmission risk than cesarean section, which fits the ascending-infection model.

Maternal colonization does not automatically translate into adverse outcomes. Whether it stays benign or progresses to ascending intrauterine infection depends on cervical competence, the virulence of the infecting serovar, bacterial load, co-colonization with organisms such as *Mycoplasma hominis*, changes in the vaginal microbiome associated with bacterial vaginosis, and the character of the maternal and fetal immune response [12,13]. Ireland and Keelan’s model captures this well: vaginal colonization without ascending infection carries low risk; ascending infection with an attenuated inflammatory response carries moderate risk; and ascending infection paired with a robust pro-inflammatory response carries the highest risk of adverse pregnancy outcome [12]. This helps explain why, despite how common maternal colonization is, only a subset of pregnancies go on to develop clinically significant intrauterine infection.

## 6. Intrauterine Infection, Chorioamnionitis, and Preterm Birth

*Ureaplasma* spp. are the microorganisms most often found in the amniotic fluid and placentas of women who deliver preterm, and their detection there is consistently linked to histological chorioamnionitis and adverse pregnancy outcomes [9,10,53]. The process typically begins with ascending colonization from the cervicovaginal space into the choriodecidual space, sometimes weeks or months before any clinical symptoms appear. Primate choriodecidual inoculation studies have since shown that early *U. parvum* infection activates the NLRP3 inflammasome within the fetal membranes, producing IL-1β-mediated inflammation even before intra-amniotic infection is established—a finding that has substantially revised the accepted timeline of pathogenesis [42].

Once inside the amniotic cavity, the organism’s surface MBA lipoproteins engage TLR2/6 and TLR9 on immune cells in the fetal membranes, choriodecidua, and amniotic epithelium, activating NF-κB and driving production of IL-1β, IL-6, IL-8, TNF-α, and matrix metalloproteinases [34,35,54]. This cascade stimulates prostaglandin E2 and F2-α synthesis via cyclooxygenase-2 upregulation, directly triggering uterine contractions and cervical ripening [9,55], while MMP release degrades the extracellular matrix of the fetal membranes and leads to premature rupture [9,56].

A systematic review and meta-analysis of 22 studies confirmed a significant link between vaginal and amniotic fluid *Ureaplasma* spp. and preterm birth [10]. A separate meta-analysis of adverse pregnancy and birth outcomes found that maternal *Ureaplasma* exposure significantly raised the risk of preterm birth, chorioamnionitis, and premature rupture of membranes [49]. Interestingly, the cytokine response *Ureaplasma* spp. provoke is milder than that seen with group B Streptococcus or *Escherichia coli*, and immune activation is blunted in preterm compared with term fetuses [40,57]. This relative immunological quiet may explain why intrauterine colonization can persist asymptomatically for weeks before finally triggering delivery—a pattern well documented in clinical series where mothers of preterm infants colonized early in gestation show few overt signs of infection [40].

In the sheep model of chronic intrauterine *U. parvum* infection, it is the duration of exposure—not simply its presence—that determines how severe the fetal lung inflammation, epithelial necrosis, and elastin deposition and fibrosis become [58]. Placental cultures from spontaneous preterm births before 32 weeks showed histological chorioamnionitis in 83% of *Ureaplasma*-positive cases, versus 30% of *Ureaplasma*-negative cases [41]. A large prospective multicenter study confirmed that early-pregnancy vaginal colonization was significantly associated with severe intraventricular hemorrhage (10.4% vs. 2.6%), retinopathy of prematurity (21.7% vs. 10.3%), and adverse psychomotor outcomes (24.3% vs. 1.8%) among infants born before 33 weeks [17].

This inflammatory dialogue runs both ways. Elevated amniotic fluid IL-6, common in *Ureaplasma*-infected pregnancies, has been independently linked to neonatal periventricular leukomalacia and intraventricular hemorrhage [59]. Fetal inflammatory response syndrome—marked by elevated fetal cytokines and inflammatory infiltration of the umbilical cord vessels (funisitis)—occurs more often in *Ureaplasma*-infected pregnancies than in those infected with other bacteria [53], and its presence has independently been tied to neonatal white matter disease.

## 7. Neonatal Morbidities: The Multisystem Impact of Ureaplasma Colonization

### 7.1. Bronchopulmonary Dysplasia

The link between *Ureaplasma* respiratory colonization and bronchopulmonary dysplasia (BPD) is the most extensively studied of all its clinical associations, and it has held up consistently across three decades of independent meta-analyses. Three separate groups first reported the association in 1988. A 1995 meta-analysis of seventeen studies confirmed it, and a later meta-analysis of 36 cohort studies covering roughly 3000 preterm infants confirmed the link for BPD defined both as oxygen dependence at 28 days (*p* < 0.001) and at 36 weeks postmenstrual age (*p* < 0.001) [28]. A more recent meta-analysis of twelve studies (2024) found that *U. urealyticum* infection carried a pooled odds ratio of 2.30 (95% CI: 1.65–3.20) for BPD, a result that held up under sensitivity analysis and trial sequential analysis alike [15].

The underlying mechanism is increasingly clear. *Ureaplasma* spp. stimulate pulmonary cells and macrophages in vitro to produce TNF-α, IL-1β, IL-8, monocyte chemoattractant protein-1, and TGF-β1, and tracheal aspirate levels of these cytokines are already elevated in the first week of life in infants who later develop BPD [11]. Lungs from infants who died with *Ureaplasma* pneumonia show moderate to severe fibrosis, excess myofibroblasts, disordered elastin, and abundant TNF-α- and TGF-β1-immunoreactive cells—changes reproduced in the 125-day immature baboon model of antenatal *U. parvum* infection followed by postnatal ventilation [58,60]. Antenatal inflammatory priming combined with postnatal volutrauma and hyperoxia appears to generate the amplified, dysregulated inflammatory response characteristic of ‘new BPD’ [11,60].

Respiratory colonization rates rise sharply as gestational age falls: in one cohort, 65% of infants below 26 weeks were culture- or PCR-positive within the first month of life, compared with 31% of infants at or beyond 26 weeks [19]. A prospective study of infants under 32 weeks confirmed that *Ureaplasma* colonization was significantly associated with BPD only among those ventilated for five days or more—not among those ventilated more briefly—pointing to a synergy between infection and ventilator-induced injury [61].

### 7.2. Intraventricular Hemorrhage

Intraventricular hemorrhage (IVH) is one of the most feared complications of extreme prematurity, and its relationship with *Ureaplasma* colonization has drawn substantial research attention. A meta-analysis of eight studies found a pooled odds ratio of 1.62 (95% CI: 1.23–2.13) for *U. urealyticum* infection and IVH, confirmed by trial sequential analysis with no meaningful statistical heterogeneity (I^2^ = 13.7%) [15]. Individual cohort studies point the same direction: Viscardi and colleagues found that serum PCR positivity conferred a 2.5-fold increase in severe IVH (≥Grade III) after adjusting for gestational age, and Kasper and colleagues reported that in utero exposure was independently associated with both higher BPD and IVH rates [62].

That said, several single-center prospective studies have not confirmed an association between *Ureaplasma* colonization and IVH or periventricular leukomalacia [52,63]. Differences in sampling sites, detection methods, gestational age distributions, and the frequent failure to distinguish between the two *Ureaplasma* species likely explain much of this inconsistency. Biological plausibility, however, remains strong. In vitro, *Ureaplasma* spp. appear to compromise blood–brain barrier integrity by upregulating ACKR3, CXCR4, and VEGF in human brain microvascular endothelial cells, increasing paracellular permeability [43], while also inducing apoptosis in these cells through caspases 3, 7, and 9—even as they paradoxically suppress the pro-inflammatory caspases 1 and 4 [44]. This endothelial and blood–brain barrier evidence should be interpreted as supporting a contributory role for *Ureaplasma*-driven neurovascular dysfunction, not as establishing a direct causal pathway to IVH. The dominant, well-established cause of IVH in extremely preterm infants remains the intrinsic fragility of the germinal matrix microvasculature, compounded by coagulation immaturity, incomplete cerebral autoregulation, and fluctuating cerebral blood flow—mechanisms that operate independently of any infectious trigger. *Ureaplasma*-associated cytokine release and blood–brain barrier compromise most plausibly act as an additional, modifiable insult layered onto this pre-existing anatomical and physiological vulnerability, rather than as a sufficient cause on their own; the clinical epidemiology summarized above (odds ratios in the 1.6–2.5 range) is consistent with a contributory rather than a dominant effect size.

### 7.3. Neonatal Sepsis and Invasive Disease

*Ureaplasma* spp. can spread hematogenously and have been recovered from cord blood, venous blood, and cerebrospinal fluid in preterm neonates. Meta-analysis confirms a significant association between *U. urealyticum* infection and late-onset neonatal sepsis, with a pooled odds ratio of 1.54 (95% CI: 1.11–2.13) and no measurable heterogeneity (I^2^ = 0%) [15]. A large epidemiological study found that 8.4% of *Ureaplasma*-positive neonates had at least one co-infecting pathogen, compared with 4.4% of *Ureaplasma*-negative neonates—suggesting that the organism may induce immune dysregulation that opens the door to secondary infection [18]. In the sheep model, chronic fetal exposure to *U. parvum* suppresses innate immune responses in a manner resembling endotoxin tolerance [57], and a prospective cohort study linked perinatal *Ureaplasma* exposure to a higher risk of late-onset sepsis and imbalanced cytokine responses [61]. This diagnostic blind spot is especially relevant to the growing problem of culture-negative early-onset sepsis. Vasilescu and colleagues, in a recent multicenter analysis, found that a substantial share of neonates with early-onset sepsis had negative blood cultures despite clear clinical and biochemical evidence of systemic infection, and identified gestational age, prolonged rupture of membranes, and elevated acute-phase reactants as independent risk factors for culture-negative disease [64]—precisely the scenario in which *Ureaplasma* spp., invisible to standard blood culture, may be the missing cause.

### 7.4. Necrotizing Enterocolitis

The relationship between *Ureaplasma* spp. and necrotizing enterocolitis (NEC) is still unsettled. One prospective cohort study found a 2.0-fold increase in NEC incidence among *Ureaplasma*-positive infants below 33 weeks, rising to 3.3-fold below 28 weeks, alongside elevated cord blood IL-6 and IL-1β [65]. In the sheep intrauterine infection model, fetal intestinal exposure to *U. parvum* produced intestinal inflammation, reduced enterocyte proliferation, and IL-1-mediated villous atrophy [66]. Yet a meta-analysis of eight studies found a pooled odds ratio for NEC of only 1.33 (95% CI: 0.91–1.95), which did not reach significance (*p* = 0.137), and trial sequential analysis suggests the evidence threshold has not yet been crossed [15]. Differences in gestational age, birth weight, gut microbiome composition, feeding practices, and diagnostic criteria for NEC across studies likely account for much of this discordance.

### 7.5. Neuroinflammation and Neurodevelopmental Outcomes

Neuroinflammation has become a central focus of recent *Ureaplasma* research. Over the past 45 years, 35 cases of neonatal meningitis caused by *Ureaplasma* spp. or *Mycoplasma hominis* have been described, with the most immature preterm infants at greatest risk [67]. Reported presentations range from sepsis-like syndromes, apnea, and seizures to internal hydrocephalus and lasting neurodevelopmental impairment—including one documented case of chronic *Ureaplasma parvum* meningitis lasting eight months in a former preterm infant [45].

The American Academy of Pediatrics’ Red Book acknowledges real diagnostic and therapeutic uncertainty around *Ureaplasma* CNS infections, noting reports of preterm infants with *Ureaplasma* species recovered from cerebrospinal fluid regardless of whether they received antimicrobial therapy, and regardless of whether CSF sterilization was later documented [68]. This captures the central clinical paradox well: even when the organism is eradicated microbiologically, neurological sequelae—hydrocephalus, white matter injury, lasting neurodevelopmental impairment—may still occur, suggesting that the inflammatory damage is set in motion before, or independently of, the organism’s persistence. In other words, CSF sterilization should be interpreted cautiously as a therapeutic endpoint, and prevention efforts may be better directed at the antenatal inflammatory cascade itself rather than relying on postnatal microbiological clearance alone.

A prospective cohort study of infants born between 23 and 33 weeks found that intrauterine *Ureaplasma* infection was associated with cerebral palsy and lower psychomotor development scores on the Bayley Scales at 2 years corrected age [69]. In a murine model of intrauterine *U. parvum* infection, researchers observed microglial activation, delayed myelination, and disturbed neuronal development in the fetal and neonatal brain [70], and primate models showed disturbed brain growth and maturation on MRI following intra-amniotic exposure [71].

Three mechanisms appear to underlie *Ureaplasma*-driven neuroinflammation: disruption of blood–brain barrier integrity through endothelial apoptosis and upregulation of permeability-promoting receptors; suppression of local CNS immune defenses, including downregulated inflammatory caspases and blunted chemokine responses; and systemic inflammatory activation that can injure the brain through circulating cytokines even without direct CNS invasion [43,44]. The fact that *Ureaplasma* spp. can simultaneously damage the blood–brain barrier and dampen local inflammatory clearance may explain why chronic CNS infections with this organism so often occur without the classical CSF pleocytosis seen with other bacterial pathogens [67,72].

### 7.6. Retinopathy of Prematurity and Other Complications

Beyond these major morbidities, *Ureaplasma* respiratory colonization has also been linked to retinopathy of prematurity, with colonized infants showing significantly higher rates of severe disease than uncolonized peers (52% vs. 30.4% in one prospective cohort of infants ≤1250 g)—possibly through a mechanism involving reduced VEGF and increased vascular fragility [73,74]. Large epidemiological series have also confirmed associations with neonatal respiratory failure and respiratory distress syndrome, though not with pneumonia or NEC [18].

## 8. Diagnostic Approaches: Culture, PCR, and the Challenge of Clinical Relevance

Diagnosing *Ureaplasma* infection is difficult for technical, biological, and clinical reasons all at once. Culture remains the reference standard and the only method for antimicrobial susceptibility testing, but it requires specialized media (10B broth and A8 agar), strict anaerobic conditions, and prolonged incubation [32]. Its sensitivity is also poor: one study found that culture missed more than 90% of PCR-positive specimens from genital tract tissue [48].

PCR is far more sensitive, detecting fewer than 100 genome copies and picking up non-viable organisms, which is why it is now the preferred method for clinical purposes [32]. Real-time quantitative PCR enables measurement of bacterial load, which correlates in a dose-dependent manner with the intensity of the intra-amniotic inflammatory response [75]. Metagenomic next-generation sequencing (mNGS) is an emerging alternative of particular relevance here: unlike targeted PCR, it sequences all microbial genomes in a sample without prior culture or a pathogen-specific hypothesis, sidestepping both the poor sensitivity of culture and PCR’s inability to characterize polymicrobial communities at species and serovar resolution simultaneously. mNGS has already been used to detect *Ureaplasma* spp. in the gastric fluid of neonates with respiratory distress and chorioamnionitis, and its use in diagnosing neonatal infection more broadly is expanding [76]. As sequencing costs continue to fall, applying it to tracheal aspirates, amniotic fluid, and cord blood in prospective cohorts could offer an unprecedented view of the microbiological landscape at the most vulnerable point in early life, and might finally settle whether serovar-specific virulence differences actually change clinical outcomes.

Sample type matters as much as assay choice. Vaginal and cervical swabs reflect lower genital tract colonization, which is common and, by itself, of limited prognostic value; amniotic fluid and placental or chorioamniotic tissue better reflect ascending intrauterine infection and correlate more closely with chorioamnionitis and preterm birth [9,10]; and, in the neonate, tracheal aspirate or lower respiratory tract sampling carries far greater prognostic weight for bronchopulmonary dysplasia than nasopharyngeal sampling alone, reflecting site-specific rather than generic colonization risk [19]. Turnaround time differs sharply between methods: culture requires specialized media and days to weeks of incubation before a result is available, which is clinically unworkable for time-sensitive decisions such as whether to treat evolving chorioamnionitis or neonatal sepsis, whereas PCR can return a result within hours [32]. Cost-effectiveness has not been formally studied for *Ureaplasma*-specific screening, but the reagent and labor costs of routine PCR are modest compared with the downstream costs of unrecognized bronchopulmonary dysplasia, intraventricular hemorrhage, or late-onset sepsis—an argument that merits formal economic modeling rather than assumption. False positives are less an assay artifact than an interpretive one: because PCR detects non-viable organisms and residual DNA, a positive result does not by itself distinguish active infection from cleared or historical colonization, which is why quantitative bacterial load and repeat testing, rather than qualitative positivity alone, are increasingly proposed as the more clinically meaningful readout [75].

Whether a positive result—by culture or PCR—is clinically meaningful remains the most contested question in the field. Abe and colleagues, examining gastric fluid from 47 NICU admissions, found *Ureaplasma* positivity in 19% of cases (all *U. parvum*), and noted a striking difference by gestational age: preterm infants showed markedly elevated IgM consistent with prolonged intrauterine exposure, while term infants showed prominent maternal inflammatory signs, including non-reassuring fetal status and elevated CRP—suggesting the immune consequences of exposure differ qualitatively depending on when in gestation it occurs [40].

Interpreting a positive *Ureaplasma* test is complicated further by the fact that macrolide antibiotics have both antimicrobial and anti-inflammatory effects. The most rigorous randomized trial of intravenous azithromycin in *Ureaplasma*-colonized preterm infants successfully eradicated the organism from the respiratory tract but failed to significantly reduce the primary composite outcome of BPD or death at 36 weeks postmenstrual age [16]. A smaller, single-center trial of clarithromycin, by contrast, did report a reduction in BPD [21]—raising the possibility that immunomodulatory rather than purely antimicrobial effects may be responsible for any clinical benefit observed. This mismatch between microbiological eradication and clinical outcome makes it hard to establish a causal pathogenic role for *Ureaplasma* from antibiotic trials alone, and it underscores the need for adequately powered studies that can tease apart antimicrobial from anti-inflammatory mechanisms.

## 9. Therapeutic Approaches: Macrolides, Clinical Trials, and Remaining Uncertainties

Because *Ureaplasma* spp. lack a cell wall, they are intrinsically resistant to β-lactams, aminoglycosides, and glycopeptides, which leaves only agents that inhibit protein synthesis or topoisomerases—macrolides, tetracyclines, fluoroquinolones, and chloramphenicol [4,32]. In neonatal practice, macrolides are the preferred class; tetracyclines are generally avoided in young neonates because of dental and bone toxicity, except in specific circumstances such as *Ureaplasma*-positive CSF, and fluoroquinolones raise theoretical concerns about cartilage development [4].

*Ureaplasma* spp. are susceptible to erythromycin in vitro and its pharmacokinetics are favorable, yet trials of erythromycin therapy in colonized preterm infants have consistently failed to prevent BPD or eradicate respiratory colonization [11,77]. Attention has since shifted to azithromycin and clarithromycin, which offer better in vitro activity, stronger anti-inflammatory properties, and more favorable pharmacokinetics in preterm infants.

The macrolide class exerts two mechanistically distinct effects that are easy to conflate when interpreting trial results. The antimicrobial effect—inhibition of the 50S ribosomal subunit, halting bacterial protein synthesis—is what eradicates *Ureaplasma* from the respiratory tract or amniotic cavity, and it is the effect captured by microbiological cure. The anti-inflammatory effect operates largely independently of bacterial killing: macrolides suppress NF-κB activation, downregulate pro-inflammatory cytokine transcription, and reduce neutrophil chemotaxis in a manner that can persist even against macrolide-resistant or cell-wall-deficient organisms. This distinction has direct trial design implications. A trial powered only to demonstrate microbiological eradication, as the pivotal azithromycin phase IIb trial was, can succeed on that endpoint while still failing to show a difference in a clinical outcome such as BPD if the relevant injury is driven by an anti-inflammatory rather than a purely antimicrobial pathway—precisely the dissociation observed in that trial [16]. Conversely, a clinical benefit observed without confirmed eradication, as in the smaller clarithromycin trial, is more consistent with an anti-inflammatory than a purely antimicrobial mechanism [21]. Outside the *Ureaplasma* literature, non-antibacterial macrolide derivatives that retain anti-inflammatory activity while lacking antimicrobial activity (for example, the EM900 series developed from erythromycin) have been investigated in chronic airway inflammatory disease specifically to separate these two effects pharmacologically; whether such compounds have any role in *Ureaplasma*-associated neonatal disease is untested and remains speculative, but the concept underscores that future *Ureaplasma* trials should consider co-primary microbiological and inflammatory (e.g., cytokine) endpoints rather than eradication alone.

A phase I pharmacokinetic study of single-dose IV azithromycin at 10 and 20 mg/kg in mechanically ventilated preterm neonates (24–28 weeks) suggested that repeated 20 mg/kg doses might achieve a favorable AUC/MIC90 ratio, while 10 mg/kg was inadequate for clearing the organism [20]. The subsequent phase IIb randomized placebo-controlled trial by Viscardi and colleagues—the most methodologically rigorous study to date—confirmed that IV azithromycin effectively eradicates respiratory *Ureaplasma* in infants born before 29 weeks [16]. Even so, the primary composite outcome of BPD or death did not differ significantly between groups, a result that held at two-year follow-up of pulmonary and neurodevelopmental outcomes [22].

A single-center randomized trial of clarithromycin did document a significant BPD reduction in *U. urealyticum* positive preterm infants, with a lower rate of BPD at 36 weeks postmenstrual age among treated infants than among those given placebo and confirmed microbiological eradication [21]. This result deserves the same methodological scrutiny applied above to the larger azithromycin trial: it comes from a single center, enrolled a comparatively small number of infants, and has not been independently replicated in a multicenter setting, so it should be regarded as hypothesis-generating rather than practice-changing until a larger trial confirms or refutes it—precisely the same standard by which the negative primary outcome of the azithromycin phase IIb trial should be read as inconclusive rather than definitively negative, given that trial’s own power limitations for the composite BPD-or-death endpoint. In primate models, maternal azithromycin for intra-amniotic *Ureaplasma* infection delayed preterm delivery, reduced fetal lung injury, and improved fetal hemodynamics—compelling proof of concept for prenatal intervention [23,78]. For the rare but serious complication of *Ureaplasma* meningitis, case reports describe successful use of chloramphenicol, tetracyclines, and fluoroquinolones [45,67,79].

At present, there is no phase III evidence that treating *Ureaplasma* respiratory colonization improves outcomes, so routine treatment in preterm neonates is not standard of care. Standard antibiotic regimens for preterm premature rupture of membranes likewise fail to eradicate *Ureaplasma* spp. from the amniotic cavity [4,16]. The therapeutic gap is real, and the field is waiting on adequately powered trials to close it.

## 10. The Host–Pathogen Interface: Why Some Colonized Individuals Progress to Disease

The central puzzle of *Ureaplasma* biology—near-universal colonization alongside relatively selective harm—is best understood through the lens of host-pathogen interaction. Ireland and Keelan, reviewing the historical literature on anti-*Ureaplasma* antibodies, found that culture-positive amniotic fluid combined with elevated anti-*Ureaplasma* serum IgG was associated with a markedly higher odds of adverse pregnancy outcome (OR 81.00 at genetic amniocentesis), whereas colonization without a detectable antibody response did not predict adverse outcome nearly as strongly [12].

Gestational age at the time of infection also shapes the quality and magnitude of the inflammatory response: preterm fetuses mount lower cytokine responses to *Ureaplasma* than term fetuses do [57]. This dependence on gestational age helps explain why many women colonized early in pregnancy remain asymptomatic for weeks or months before delivering prematurely, while those colonized closer to term show more obvious signs of acute infection [40].

Translating the concept of conditional pathogenicity into clinical practice requires testable, quantifiable criteria rather than a binary colonized/not-colonized framework. Three candidate criteria are already supported by existing data. First, bacterial load: real-time quantitative PCR shows that amniotic fluid *Ureaplasma parvum* burden correlates in a dose-dependent fashion with the intensity of the intra-amniotic inflammatory response, suggesting a threshold effect worth defining prospectively [75]. Second, serovar identity: *U. parvum* serovar 3 has shown the most consistent association with spontaneous preterm birth across prospective cohorts, raising the possibility of a serovar-based risk score once larger series confirm the pattern [47]. Third, host response: the combination of culture-positive amniotic fluid with elevated anti-*Ureaplasma* serum IgG predicts adverse pregnancy outcome far more strongly than colonization status alone (odds ratio 81.00 in the historical cohort analyzed by Ireland and Keelan), indicating that a composite microbiological-plus-serological marker may outperform either alone [12]. None of these criteria have yet been validated in a prospective, adequately powered cohort, but each offers a concrete, measurable variable that future studies could use to move the field from a qualitative “commensal-or-pathogen” debate toward a quantitative risk model.

Polymicrobial interactions matter too. Co-colonization of the vagina with *Ureaplasma* spp. and *Mycoplasma hominis* is associated with worse pregnancy outcomes than colonization with either organism alone [80], and bacterial vaginosis is itself an independent risk factor for preterm birth that appears to amplify *Ureaplasma*-related risk when the two coexist [12].

Serovar-specific virulence is still an active research question—*U. parvum* serovar 3 has the most consistent association with spontaneous preterm birth across prospective studies [47]. There is also the possibility that *Ureaplasma* spp. can induce a state of immune tolerance, similar to endotoxin tolerance in sepsis, that paradoxically protects the fetus from a devastating fetal inflammatory response at the cost of impaired pathogen clearance and ongoing low-grade inflammation. In the sheep model, chronic intrauterine *U. parvum* exposure blunted innate immune responses upon re-challenge, consistent with a protective preconditioning effect [57,72].

## 11. Clinical Implications: Toward a New Screening and Management Paradigm

The accumulated evidence justifies rethinking where *Ureaplasma* spp. fit into obstetric and neonatal practice. In obstetrics, screening should be considered for pregnancies at elevated risk of preterm birth—a history of preterm delivery, short cervix, bacterial vaginosis, or preterm premature rupture of membranes. A randomized trial of antenatal azithromycin in culture- or PCR-positive women with short cervix or prior preterm birth, modeled on the primate data showing pregnancy prolongation and reduced fetal lung injury [23,78], would be the most scientifically justified trial design available—and it is conspicuously missing from the current clinical trials landscape.

In neonatal medicine, universal or targeted PCR screening of extremely preterm infants for *Ureaplasma* spp. during the first week of life is now defensible on prognostic grounds alone: colonization status identifies infants at meaningfully higher risk of BPD, IVH, and late-onset sepsis, who might benefit from closer respiratory monitoring, protective ventilation strategies, and enrollment in therapeutic trials. The finding that lower respiratory tract colonization in mechanically ventilated infants carries a 7.9-fold higher risk of moderate-to-severe BPD than nasopharyngeal colonization alone [19] shows just how much distinguishing colonization site actually matters clinically.

Eradicating *Ureaplasma* spp. from the intrauterine or neonatal respiratory environment before the organ injury cascade becomes irreversible—rather than simply managing its consequences afterward—is the most intellectually coherent strategy, even though it remains clinically unproven. Educating perinatal teams about this growing evidence base, paired with institutional protocols for PCR-based screening in defined high-risk groups, is a necessary first step toward closing the diagnostic and therapeutic gap (Table 4).

## 12. Discussion

The history of *Ureaplasma* research shows just how hard it is to pin causality on an organism this common. For decades, it was dismissed as an innocent bystander, its ubiquity in healthy women read as biological neutrality rather than as a sign of conditional pathogenicity. The careful but inconclusive infertility studies of Idriss, Rehewy, and their contemporaries could not settle whether elevated culture rates reflected cause or coincidence—a stalemate rooted largely in methodology: insensitive culture-based detection, no serovar discrimination, and, until 2002, no formal taxonomic distinction between *U. urealyticum* and *U. parvum* at all [29].

The shift in understanding came not from any single landmark trial but from the convergence of animal models, molecular epidemiology, and meta-analytic synthesis. The links to bronchopulmonary dysplasia, intraventricular hemorrhage, and late-onset sepsis have now crossed the threshold of statistical certainty, with consistent effect sizes across independent datasets [15]. The evidence for necrotizing enterocolitis is weaker—most likely a consequence of diagnostic heterogeneity and insufficient statistical power—but animal data showing intestinal inflammation and villous atrophy still provide enough biological plausibility to justify continued investigation [15,66].

What remains genuinely unresolved is how to translate this into treatment. The most rigorous randomized trial to date confirmed that IV azithromycin effectively eradicates respiratory *Ureaplasma* colonization, yet it did not reduce the composite outcome of BPD or death [16]. This gap between microbiological success and clinical benefit matters a great deal: it may mean that, by the time postnatal treatment starts, the inflammatory cascade driving organ injury is already irreversible, or that the critical window of exposure is antenatal rather than postnatal. The smaller clarithromycin trial that did show clinical benefit [21] raises the possibility that immunomodulatory rather than purely antimicrobial effects are doing the work—a distinction that complicates how every macrolide study in this field should be read.

So why does the ‘missing villain’ persist in everyday practice despite decades of accumulating evidence? Four barriers explain the gap, and they are as much cognitive as they are scientific. Diagnostic invisibility is one: standard culture misses more than 90% of PCR-positive specimens, so the organism goes systematically undetected in routine sepsis workups and amniotic fluid cultures [48]. Taxonomic conflation is another—older studies that never distinguished *U. urealyticum* from *U. parvum* obscured real differences in serovar-specific virulence, creating a false impression of a single, homogeneous commensal [29]. A third is the therapeutic gap: the antibiotics typically used for preterm premature rupture of membranes, ampicillin and azithromycin, do not actually eradicate *Ureaplasma* from the amniotic cavity, a failure rarely acknowledged in day-to-day practice [4,16]. Finally, and perhaps most stubbornly, there is the legacy of the commensalist paradigm: because the organism colonizes 40–80% of healthy adults without obvious harm, biological harmlessness became the default assumption [5]—an anchor that the accumulated evidence has struggled to dislodge.

The model that best fits all of this evidence is one of conditional pathogenicity: *Ureaplasma* spp. typically coexist with their host without clinically significant consequence, yet can cause real harm once a threshold combination of gestational vulnerability, bacterial burden, serovar virulence, and host immune competence is crossed [12,15]. That conditionality is not evidence of harmlessness—it is evidence of biological complexity. The distinction is analogous to *Staphylococcus aureus* on human skin: the organism colonizes a large proportion of healthy adults without incident, yet under the right conditions—a breach in the epithelial barrier, an immunocompromised host, a sufficient inoculum—the same organism causes invasive, life-threatening disease. Colonization and infection are different biological states produced by the same organism, and the fact that most colonized individuals never progress to disease no more clears *Ureaplasma* of a causal role in the minority who do than the ubiquity of asymptomatic *S. aureus* carriage clears it of responsibility for invasive staphylococcal disease. The right clinical response, then, is not to dismiss the organism but to stratify risk, target diagnostics appropriately, and develop evidence-based protocols that can distinguish antimicrobial from anti-inflammatory mechanisms of action.

## 13. Future Directions

The next decade of *Ureaplasma* research needs to tackle several open questions with a rigor that matches their clinical stakes. Three priorities stand out as both urgent and scientifically ready to pursue.

Adequately powered randomized interventional trials: The Viscardi phase IIb trial proved that IV azithromycin achieves microbiological eradication, but it was not powered to detect a clinical effect on BPD or death [16]. A phase III multicenter trial—enrolling infants below 28 weeks, stratified by lower respiratory tract colonization status and serovar, and powered for BPD at 36 weeks postmenstrual age as the primary endpoint—is now both scientifically justified and, arguably, ethically overdue. Antenatal trial designs targeting PCR-positive women with a short cervix or prior preterm birth, who would receive azithromycin in the second trimester, are supported by strong primate data [23,78] and represent a largely unexplored avenue that could meaningfully reduce the burden of extreme prematurity. Prospective registries of extremely preterm infants with systematic *Ureaplasma* PCR testing at enrollment would also help, providing the observational infrastructure needed to identify the most vulnerable subgroups, characterize the natural history of colonization in contemporary cohorts, and counteract the publication bias that currently favors positive findings.

Metagenomic next-generation sequencing for clinical diagnostics. Given that culture misses more than 90% of PCR-positive specimens [48], and given how much species- and serovar-level resolution matters for assessing differential virulence, mNGS looks like a genuinely transformative candidate for *Ureaplasma* diagnostics. Unlike targeted PCR, it characterizes the entire microbial community in a sample of amniotic fluid, placental tissue, tracheal aspirate, or blood all at once—detecting *Ureaplasma* spp. alongside polymicrobial co-colonizers such as *Mycoplasma hominis* and identifying the specific serovars present without needing a prior hypothesis. Applying it to neonatal gastric fluid, tracheal aspirates, and cord blood could finally answer whether the serovar-specific virulence differences seen for *U. parvum* serovar 3 and certain *U. urealyticum* serovars actually translate into different clinical outcomes at the bedside.

Serovar-stratified prospective cohort studies. The field’s persistent failure to distinguish *U. urealyticum* from *U. parvum* at the serovar level remains the single biggest obstacle to resolving the question of conditional pathogenicity. Future studies should make real-time quantitative PCR with species- and serovar-level discrimination the standard, enabling dose–response analyses of bacterial load, serovar identity, and outcome severity. Pairing this with host genomic data—particularly TLR2 and TLR9 polymorphisms known to modulate inflammatory responses to *Ureaplasma* lipoproteins—could support individualized risk stratification and move the field closer to precision medicine in perinatal care.

## 14. Conclusions

*Ureaplasma urealyticum* and *Ureaplasma parvum* can no longer reasonably be treated as clinically inert commensals in the perinatal population. Based on evidence-based medicine, we are now confident that *Ureaplasma* colonization significantly raises the risk of bronchopulmonary dysplasia, intraventricular hemorrhage, and late-onset neonatal sepsis in preterm infants, and increasingly confident that it contributes to chorioamnionitis, preterm birth, neurodevelopmental delay, and retinopathy of prematurity. The underlying mechanisms—TLR-mediated cytokine activation, blood–brain barrier disruption, prostaglandin-driven preterm labor, and immune modulation—are now molecularly well-defined and experimentally supported.

Yet translating this evidence into practice remains unfinished business. Screening is not routine, treatment protocols do not exist, and the therapeutic gap persists. The ‘missing villain’ in this review’s title reflects a clinical community that has not yet acted on a substantial and growing body of evidence—partly because of diagnostic complexity, partly because phase III therapeutic trials are still lacking, and partly because the old commensalist paradigm has proven hard to shake.

The case for action is clear. Pregnancy monitoring guidelines should incorporate *Ureaplasma* screening for high-risk pregnancies. Neonatal intensive care units should consider PCR-based screening of extremely preterm infants. And adequately powered randomized trials of antenatal and postnatal azithromycin—targeting the populations identified here as most vulnerable—are urgently needed. The age of innocence for *Ureaplasma* spp. is over. What the evidence now calls for is a matching clinical response by the obstetrical and neonatal communities. This response should include risk-stratified screening, targeted diagnostics, and interventional trials large enough to finally tell us whether early identification and treatment can reduce the burden of prematurity-related morbidities in an at-risk population.

## Figures and Tables

**Table 1:** Key molecular pathways activated by *Ureaplasma* spp.: receptors, adaptors, effectors, and tissue outcomes.

Pathway	Receptor/Trigger	Adaptor/Signal	Key Effectors	Tissue Outcome
TLR2/6-NF-κB	MBA lipoprotein (diacylated); TLR2/6 heterodimer	MyD88 → IRAK4/IRAK1 → TRAF6 → IKK → IκBα degradation → NF-κB (p65/p50) nuclear translocation	IL-1β, IL-6, IL-8, TNF-α, COX-2, MMP-1/8/9	Chorioamnionitis; fetal membrane degradation; preterm labor via PGE2/PGF2-α
TLR9-NF-κB	Unmethylated CpG DNA (released during replication)	MyD88 → IRAK4 → TRAF6 → NF-κB; parallel MAPK (ERK1/2, p38, JNK)/AP-1 axis	Amplified IL-6, IL-8, TNF-α; AP-1-driven cytokine transcription	Amplified choriodecidual inflammation; sustained fetal inflammatory response syndrome
NLRP3 Inflammasome/Pyroptosis	K^+^ efflux; mitochondrial ROS; lysosomal damage from phagocytosed *Ureaplasma*	NLRP3–ASC–procaspase-1 assembly → caspase-1 activation → gasdermin D cleavage	Mature IL-1β, IL-18; DAMP release via pyroptotic pore formation	Amplified sterile inflammation post-clearance; preterm birth; BPD priming; IVH substrate
BBB Disruption	HBMEC ACKR3/CXCR4 upregulation; VEGF induction	Claudin-5/occludin transcriptional downregulation; MMP-2/9 release; intrinsic caspase cascade (caspases 3, 7, 9)	Paracellular permeability ↑; HBMEC apoptosis; paradoxical suppression of caspases 1/4	IVH; white matter injury; chronic CNS infection without CSF pleocytosis; neurodevelopmental impairment
Biofilm/Antibiotic Tolerance	MBA-mediated adhesion to epithelial/endotracheal tube surfaces; EPS matrix formation	Reduced antimicrobial diffusion; altered metabolic state; persister cell emergence; density-dependent MBA phase variation	Elevated MIC to azithromycin/ciprofloxacin; immune evasion; chronic intra-amniotic colonization	Treatment failure; sustained low-grade inflammation; failure of standard PPROM antibiotic regimens to eradicate organism
TGF-β1/Pulmonary Fibrosis	Macrophage/pulmonary cell activation by *Ureaplasma*; MCP-1 release	TGF-β1 → myofibroblast differentiation; elastin deposition; fibrotic remodeling; synergy with volutrauma/hyperoxia	TNF-α, IL-1β, IL-8, TGF-β1 in tracheal aspirates; disordered elastin; ↑ myofibroblasts	Bronchopulmonary dysplasia (new BPD); impaired alveolarization; ventilator-augmented lung injury

Abbreviations: MBA, multiple-banded antigen; HBMEC, human brain microvascular endothelial cells; BBB, blood–brain barrier; EPS, extracellular polymeric substance; PPROM, preterm premature rupture of membranes; BPD, bronchopulmonary dysplasia; IVH, intraventricular hemorrhage; ROS, reactive oxygen species; DAMP, damage-associated molecular pattern; MIC, minimum inhibitory concentration; COX-2, cyclooxygenase-2; MMP, matrix metalloproteinase; PGE2/PGF2-α, prostaglandin E2/F2-alpha.

**Table 2:** Summary of key clinical studies on neonatal morbidities associated with *Ureaplasma* spp. colonization.

Study (Year)	Population	Sample Size	Key Finding	OR (95% CI)
Cai et al., 2024 [15] (meta-analysis)	Preterm infants	~3000	U. urealyticum infection significantly associated with BPD, IVH, and late-onset sepsis; NEC association non-significant	BPD: 2.30 (1.65–3.20); IVH: 1.62 (1.23–2.13); Sepsis: 1.54 (1.11–2.13)
Viscardi et al., 2020 [16] (phase IIb RCT)	Preterm < 29 weeks, *Ureaplasma*-colonized	~100	IV azithromycin eradicated respiratory *Ureaplasma* colonization but did not significantly reduce BPD or death at 36 weeks PMA	BPD or death: RR 1.00 (95% CI 0.71–1.41), *p* = 1.00 (primary composite outcome non-significant; microbiological eradication confirmed)
Rittenschober-Böhm et al., 2021 [17] (prospective cohort)	Infants < 33 weeks; maternal vaginal *Ureaplasma* colonization in early pregnancy	~500	Maternal colonization associated with severe IVH (10.4% vs. 2.6%), ROP (21.7% vs. 10.3%), and adverse psychomotor outcomes (24.3% vs. 1.8%)	Severe IVH: 10.4% vs. 2.6% (*p* < 0.05); exact OR not reported in source; ROP: 21.7% vs. 10.3% (*p* < 0.05)
Ma et al., 2024 [18] (retrospective cohort)	Hospitalized neonates, all gestational ages	7257	PCR detection rate 7.73%; highest in 1–7-day age group, lower gestational age, and vaginal delivery; significant associations with BPD and respiratory failure	Not reported (prevalence study)
Viscardi et al., 2023 [19] (prospective observational)	Preterm infants < 32 weeks	Not reported	Lower respiratory tract (tracheal aspirate) colonization associated with 7.9-fold increased risk of moderate-severe BPD vs. nasopharyngeal colonization alone	RR 7.9 (95% CI 2.0–31.4) for moderate-severe BPD (lower vs. upper respiratory tract colonization)

Abbreviations: BPD, bronchopulmonary dysplasia; IVH, intraventricular hemorrhage; ROP, retinopathy of prematurity; RCT, randomized controlled trial; PMA, postmenstrual age; OR, odds ratio; CI, confidence interval; RR, relative risk; PCR, polymerase chain reaction.

**Table 3:** Key randomized controlled trials and phase studies of macrolide therapy for *Ureaplasma* spp. colonization in preterm neonates and in primate models.

Study (Year)	Design & Population	Intervention	Primary Outcome	Key Results	Interpretation
Viscardi et al., 2013 [20] (Phase I PK)	PK/safety study; mechanically ventilated preterm neonates 24–28 weeks, *Ureaplasma*-colonized	Single IV azithromycin dose 10 or 20 mg/kg	PK parameters; safety profile	20 mg/kg predicted favorable AUC/MIC90; 10 mg/kg inadequate; both doses well-tolerated	Established dosing rationale for Phase IIb trial; demonstrated safety in extreme prematurity
Ozdemir et al., 2011 [21] (Single-center RCT)	RCT; preterm infants < 32 weeks, U. urealyticum-positive tracheal aspirate; Turkey	Clarithromycin 10 mg/kg/day IV for 4 weeks vs. placebo	BPD at 36 weeks PMA	Significant BPD reduction: 30% vs. 58% (*p* = 0.02); microbiological eradication confirmed	Only RCT showing clinical BPD benefit; single-center, underpowered; requires multicenter replication
Viscardi et al., 2020 [16] (Phase IIb RCT)	Multi-center RCT; preterm < 29 weeks, *Ureaplasma*-colonized (tracheal aspirate or nasopharynx); USA	IV azithromycin 20 mg/kg single dose (±repeat doses) vs. placebo	BPD or death at 36 weeks PMA (composite)	Microbiological eradication confirmed; BPD or death RR 1.00 (95% CI 0.71–1.41), *p* = 1.00—non-significant	Key finding: dissociation between microbiological and clinical endpoints; suggests antenatal cascade may be irreversible by postnatal treatment
Viscardi et al., 2022 [22] (Phase IIb 2-yr follow-up)	Longitudinal follow-up of Viscardi 2020 cohort; survivors assessed at 2 years corrected age	Azithromycin vs. placebo (as assigned in 2020 trial)	Pulmonary and neurodevelopmental outcomes at 2 years CA	No significant differences in pulmonary or neurodevelopmental outcomes; lower respiratory tract colonization subgroup identified as highest-risk	Identifies target population for Phase III: ELGA infants with lower respiratory tract colonization
Grigsby et al., 2012 [23] (Primate RCT)	Rhesus macaque model; intra-amniotic U. parvum inoculation; *n* = 20	Maternal IV azithromycin 2 g loading + 1 g/day for 10 days vs. saline	Preterm delivery rate; fetal lung injury	Azithromycin delayed preterm delivery (*p* < 0.05), significantly reduced fetal lung injury scores and cytokine levels	Proof-of-concept for antenatal treatment window; supports design of human antenatal azithromycin RCTs

Abbreviations: RCT, randomized controlled trial; BPD, bronchopulmonary dysplasia; PMA, postmenstrual age; PK, pharmacokinetics; IV, intravenous; ELGA, extremely low gestational age; CA, corrected age; CI, confidence interval; RR, relative risk; AUC, area under the curve; MIC, minimum inhibitory concentration.

**Table 4:** Proposed risk-based screening and management pathway for *Ureaplasma* spp. in obstetric and neonatal practice, synthesized from the clinical and diagnostic evidence discussed above. This pathway reflects the authors’ interpretation of the current evidence and is not a validated clinical protocol; it is offered to organize risk-stratified screening pending the outcome of adequately powered interventional trials.

Setting	Trigger/Risk Factor	Recommended Test	Action if Positive
Obstetric (antenatal)	Prior spontaneous preterm birth, short cervix, bacterial vaginosis, or preterm premature rupture of membranes	Vaginal/cervical PCR; amniotic fluid PCR if PPROM or suspected chorioamnionitis	Risk-stratify by bacterial load and clinical signs of ascending infection; prioritize for antenatal azithromycin trials where available; manage per standard obstetric protocols outside a trial, since no eradication therapy is yet proven to improve outcomes [23,78]
Neonatal (respiratory)	Birth < 28 weeks, mechanical ventilation, or known maternal colonization/chorioamnionitis	Lower respiratory tract (tracheal aspirate) PCR in the first week of life, preferred over nasopharyngeal sampling alone	Intensify respiratory monitoring and protective ventilation; prioritize for enrollment in adequately powered interventional trials; routine antibiotic treatment is not currently supported outside research protocols [16,19]
Neonatal (culture-negative sepsis)	Clinical/biochemical signs of sepsis with negative standard blood culture, especially with prolonged rupture of membranes or elevated acute-phase reactants	Blood/CSF *Ureaplasma* PCR alongside the standard culture-negative sepsis workup	Consider *Ureaplasma* as an occult pathogen; correlate with quantitative load and clinical trajectory rather than treating a single qualitative result in isolation [64]

Abbreviations: PPROM, preterm premature rupture of membranes; PCR, polymerase chain reaction; CSF, cerebrospinal fluid.

## Data Availability

No new data were created or analyzed in this study. Data sharing is not applicable to this article.
